# Regulation of Microbiota by Vitamin D Receptor: A Nuclear Weapon in Metabolic Diseases

**DOI:** 10.11131/2018/101377

**Published:** 2018-08-09

**Authors:** Danika Bakke, Ishita Chatterjee, Annika Agrawal, Yang Dai, Jun Sun

**Affiliations:** 1 Division of Gastroenterology and Hepatology, Medicine, University of Illinois at Chicago, USA; 2 Hinsdale Central High School, 5500 S Grant St, Hinsdale, IL 60521, USA; 3 Department of Bioengineering, College of Engineering/College of Medicine, University of Illinois at Chicago, USA

**Keywords:** Inflammation, infection, bacteria, probiotics, FMT, microbiome, nuclear receptor

## Abstract

Metabolic syndrome is a multi-faceted disease. The microbiota, as a newly discovered organ, contributes to the pathogenesis and progression of metabolic syndrome. Recent studies have demonstrated that nuclear receptors play critical roles in metabolic diseases. In the current review, we discuss the general role of the microbiome in health and metabolic syndrome. We summarize the functions of the nuclear receptor vitamin D receptor (VDR) in metabolism. The focus of this review is the novel roles of vitamin D/VDR signaling in regulating inflammation and the microbiome, especially in obesity. Furthermore, we extend our discussion of potential gut-liver axis mediated by VDR signaling and microbiota in obesity. Finally, we discuss the potential clinical application of probiotics and fecal microbiota transplantation in prevention and treatment of metabolic syndrome. Insights into nuclear receptors in metabolism and metabolic diseases will allow us to develop new strategies for fighting metabolic diseases.

## 1. Introduction

Metabolic syndrome (MetS) is a challenging medical problem characterized by the association of insulin resistance (IR)/type 2 diabetes (T2DM), hyperlipidemia, hypertension, and abdominal obesity [1]. Obesity can induce chronic, low grade inflammation in adipose and other tissues, which promotes insulin resistance. The important risk factors for MetS are diet, genetics, aging, stress, sedentary behavior or low physical activity, disrupted chronobiology/sleep, mood disorders/psychotropic medication use, and excessive alcohol use. The increasing prevalence of obesity and its role in contributing to cardiovascular disease (CVD) and T2DM are fueling interest in MetS. Lifestyle changes to improve diet and exercise or gastric bypass surgery can reduce obesity and improve insulin resistance [2]. However, MetS is a multi-faceted disease, and there is no one ‘gold standard’ treatment option.

Gut bacteria have been implicated as critical regulators of host metabolism [3]. Gut microbial dysbiosis is emerging as one of the crucial risk factors for developing obesity, atherosclerosis, CVD, T2DM, and MetS [4–11]. Recent studies in both genetic and diet-induced animal models of obesity have shown that imbalance in the microbiota is related to an increased Firmicutes/Bacteroidetes phyla ratio [12, 13]. The bacteria *Clostridium coccoides, Lactobacillus reuteri, Akkermansia muciniphila, Clostridium histolyticum,* and *Staphylococcus aureus* are altered in obesity [14, 15]. The impact of the gut microbiota on host metabolism makes it a potentially viable target for treating MetS.

In the current review, we will discuss the general role of the microbiome in health and MetS, focusing on the role of the nuclear receptor vitamin D receptor in this interaction.

## 2. Vitamin D and VDR in Metabolism

1,25-dihydroxyvitamin D (l,25(OH)_2_D_3_), the active form of vitamin D, is generated in the skin after exposure to UV light or absorbed from a diet of vitamin D-rich foods. l,25(OH)_2_D_3_acts primarily through its receptor VDR. VDR is a nuclear receptor and transcription factor that directly or indirectly regulates over 900 genes [16]. VDR mediates most known functions of l,25(OH)_2_D_3_. Once VDR binds with l,25(OH)_2_D_3_,VDR heterodimerizes with the retinoid X receptor (RXR). Activated VDR then binds to the vitamin D-response element (VDRE) in the target gene promoter to regulate gene transcription. VDR is expressed in a variety of tissues, including the intestines, adipose, and liver (https://www.proteinatlas.org) [17], and modulates metabolic and immune system processes [18–22].

VDR is a critical regulator of host metabolic health. Low levels of vitamin D or inactivating polymorphisms in VDR have been associated with inflammatory and metabolic disorders [23–30]. Vitamin D deficiency causes rickets, characterized by hypoparathyroidism and impaired bone mineralization. Results from studies on mice given vitamin D-deficient diets and Vdr knockout (‘*Vdr*^*−/−*^mice) mice show the importance of vitamin D/VDR in health [20, 31–34]. Vitamin D-deficiency or loss of VDR results in inflammation, impaired adiposity, and weakened bones. Vitamin D regulates bone development and calcium homeostasis through its actions in the intestine, kidney, and bone. Vitamin D regulates calcium absorption in the intestine and resorption in the kidney by activating transcellular calcium transport, and intestine-specific Vdr knockout mice (VDR^ΔIEC^) exhibit reduced calcium absorption [35].

VDR promotes cellular health by regulating autophagy. Autophagy is critical in maintaining cellular health and protecting cells from apoptosis by degrading malfunctioning organelles and invasive bacteria. During cellular starvation, autophagy is activated to break down organelles and cell components to release needed nutrients. The protein ATG16L1 mediates autophagosome formation and is transcriptionally regulated directly by VDR. Loss of ATG16L1 or VDR expression impairs autophagy, resulting in cellular dysfunction, and has been associated with intestinal dysbiosis and inflammatory diseases, including inflammatory bowel disease (IBD) [31,36].

## 3. Gut Microbiome Greatly Affects Host Metabolism

The gut microbiome has the metabolic functions to provide the host with energy through fermentation. Bacteria break down carbohydrates indigestible by the host to increase nutrient absorption. There are several hypotheses regarding how gut microbial imbalance contributes to obesity, and many studies suggest dysbiosis in the gut microbiota causes disequilibrium in energy homeostasis that eventually leads to obesity [37–39]. Regardless, obesity, IR and MetS result as a consequence of complex interaction between immune and microbial factors with the host genetic factors [40]. Studies in both humans and mice indicate that decreased short-chain fatty acids (SCFAs) significantly contribute to the development of obesity [41–44]. Furthermore, increased levels of the bacterial endotoxin lipopolysaccharide (LPS) can promote low-grade inflammation associated with increased IR, obesity and MetS [45–47]. An abnormal increase in gram-negative bacteria, including *Enterobacter cloacae,* is associated with high levels of LPS in obesity [48]. In support, another report showed that mice fed the obesogenic ‘Western’ diet, containing high fat and low fiber, exhibit significantly increased levels of the pro-inflammatory cytokine IL-6 and endotoxemia marker LPS binding protein (LBP) in plasma. These effects were diminished remarkably after the obese mice were treated with antibiotics to suppress their gut microbiota [49]. Additionally, dysbiosis can contribute to the development of obesity by altering the production of gastrointestinal peptides related to satiety, resulting in an increased food intake and progression of disease state [50]. Interestingly, in mice, many of these metabolic disease phenotypes are transferable via feces [51]. These findings strongly implicate the vital role of microbiota in both development and advancement of metabolic conditions [52].

Microbial metabolites, including SCFAs and secondary bile acids, are taken up and affect host physiology beyond the intestine. Secondary bile acids directly stimulate host receptors farsenoid X receptor (FXR), Takeda G-protein-coupled receptor 5 (TGR5), and VDR in the intestine and liver [53]. One microbial metabolite, trimethylamine N-oxide (TMAO), is associated with diet-induced weight gain, CVD, and T2DM [54, 56, 57]. ‘Western’ diet fed mice develop an altered microbiota composition along with obesity. An obesogenic diet causes loss of microbial diversity, increased Firmicutes and Proteobacteria, and decreased Bacteroidetes populations; this diet-induced shift in the microbiome is sufficient to cause obesity. Transfer of the ‘obese’ microbiome to lean, germ-free animals induced weight gain in the recipients [40,58]. Dietary emulsifiers and artificial preservatives and sweeteners in processed food also contribute to dysbiosis and insulin resistance. Studies by Chassaing *et al* have found that obese individuals have increased microbial encroachment on the host epithelium, contributing to chronic, lowgrade inflammation and insulin resistance [57, 59]. A high fat diet alters the gut microbiota and increases bacterial LPS production and intestinal permeability, promoting inflammation, dysbiosis, and insulin resistance. These studies show that the microbiota mediates diet-induced obesity and contributes to metabolic syndrome.

Technological advances in bacterial sequencing have led to the exciting discovery that bacteria can mediate the metabolic benefits of interventions for obesity. An intriguing aspect of bariatric surgery is that insulin sensitivity improves before any significant weight loss is observed. Tremaroli *et al* have found that surgery leads to alterations in the gut microbiome that mediate at least some of the benefit of bariatric surgery [60]. Similarly, oral supplementation of the T2DM drug metformin is superior to injection partly because the drug is metabolized by gut bacteria [61]. Fecal microbiome transfer from healthy patients has been used to treat *Clostridium difficile* infection, but it has been observed that the metabolic profile of the donor can be unintentionally transmitted to the recipient [58]. These studies emphasize the critical role of the microbiome in regulating host metabolic health. This makes gut microbial modulation an attractive target in the development of treatments for obesity and metabolic disease.

## 4. VDR Regulation of Microbiome and Inflammation

Cross talk between the gut microbiome and VDR signaling affects host responses and inflammation. We have found that intestinal VDR expression regulates the host microbiome and mediates the anti-inflammatory effects of probiotics [31, 62]. Mice with intestine-specific loss of VDR expression are more susceptible to chemical models of colitis [31, 63]. *Vdr*^*−/−*^ mice are more susceptible to autoimmune diseases and have impaired differentiation of two subsets of regulatory T cells (invariant NKT and CD8αα/TCRαβ T cells) [64]. VDR signaling also promotes mucosal barrier function in the gut. Expression of the barrier protein claudin 2 is transcriptionally regulated by VDR [65]. Secretion of anti-microbial peptides such as cathelicidin and β-defensins is critical in preventing contact of gut microbes with the epithelial surface, and expression of both these AMPs is regulated by VDR [66].

VDR expression and activity in immune cells promotes tolerogenic and anti-inflammatory phenotypes. Exposing pro-inflammatory T cells from ulcerative patients to a VDR agonist is sufficient to convert them to tolerogenic regulator T cells (Tregs) [67]. Dendritic cell treated with vitamin D exhibit a tolerogenic phenotype and promote induction of Tregs [68, 69]. VDR also influences intestinal inflammation through its interaction with NF-κΒ. VDR promotes NF-κB expression but reduces intestinal inflammation by blocking NF-κΒ signaling and nuclear translocation. Loss of VDR results in loss of physical binding of NF-κBp65, reducing the level of IkappaBalpha protein, and increased transcriptional activity of NF-κB [70–73]. Interaction of VDR with NF-κΒ in macrophages suppresses the pro-inflammatory response to the bacterial endotoxin LPS [74]. Genome-wide association studies have implicated the *Vdr* gene as a mediator of inflammatory disease. A recent study found that numerous SNPs correlated with immune disorders represent binding sites for VDR and NF-κΒ, and suggests that VDR may regulate transcription indirectly through its interaction with NF-κB [75]. These data suggest the critical role of VDR in barrier functions, innate immunity and adaptive immunity.

A recent study found the direct correlation between low vitamin D levels and glucose metabolism [76]. Dietary changes (e.g., vitamin D supplementation) may reduce inflammation and ameliorate impaired insulin signaling and secretion process [76]. Vitamin D/VDR helps in maintaining healthy intestinal microbiome, thus improving glucose homeostasis in diabetes [62]. Patients with T2DM exhibit dysbiosis, and studies suggest that the microbiota plays a causative role in development of glucose intolerance and modulating the microbiota can improve glucose sensitivity [77]. VDR is a regulator of the microbiome and is also known to regulate the expression of AMPs and autophagy regulator Atgl611 [22, 31]. which are essential for microbial and intestinal homeostasis. *Vdr*^*−/−*^ mice exhibit a significant shift in the phylogenetic content of gut microbiota from their control group, including a 42% variation in β-diversity. In a human study, the shift in the microbiota was correlated with serum levels of specific bile and fatty acids, including known ligands and downstream metabolites of VDR [78]. Fecal content of *Vdr*^*−/−*^ mice shows increased Clostridium and Bacteroides populations, whereas *Lactobacillus* is depleted. In the ceacal content, *Alistipes* and *Odoribacter* were depleted, and *Eggerthella* was enriched [62]. Analysis of both human and mice gut microbiota indicated that VDR influences individual bacterial taxa such as *Parabacteroides,* which is associated with diabetes [78]. However, studies of dysbiosis and VDR in disease models are still limited. Apart from obesity and metabolic syndrome, decreased vitamin D and downregulation of VDR are also linked with the pathogenesis of several diseases associated with microbial dysbiosis, namely IBD [79–81]. cancer asthma [82, 83].

## 5. Role of Vitamin D/VDR in Obesity

Low serum vitamin D is correlated with obesity and insulin resistance [21], and many studies have identified roles for VDR in regulating metabolism. *Vdr*^*−/−*^ mice exhibit a lean phenotype and resist high fat diet-induced weight gain, including increased uncoupling protein 1 (UCP1) expression in white adipose depots resulting in energy wasting rather than storage [84]. These adipocytes, with induced UPC1 activity, increased mitochondria, and smaller lipid droplets are commonly referred to as ‘beige’ adipocytes, as they occur in white adipose depots and differentiate from white adipocyte precursors but develop brown adipocyte properties. Induction of white adipocyte trans-differentiation into metabolically active beige adipocytes is an attractive target in anti-obesity therapeutics, and loss of VDR signaling appears to be a contributing factor. On the other hand, mice with transgenic overexpression of human *Vdr* in adipocytes have increased weight gain and adiposity and decreased UCP1 expression compared to controls. Interestingly, adipocyte-specific *Vdr* knockout mice do not develop the lean phenotype observed in whole body knockout mice, suggesting that the lean phenotype is mediated by VDR signaling in other tissues, such as the intestine or liver, or caused by poor nutritional state due to impaired intestinal nutrient absorption [85]. This also indicates that vitamin D/VDR studies should include tissuespecific models.

Although total loss of VDR promotes leanness, activation of VDR signaling in the immune system may improve insulin resistance and cardiovascular aspects of MetS, including atherosclerosis. During obesity, macrophages infiltrate adipose tissue, which creates a chronically inflamed environment contributing to insulin resistance. VDR signaling in macrophages regulates their phenotype and loss of myeloid VDR promotes insulin resistance and cardiovascular disease [20]. Vitamin D deficiency exacerbates macrophage infiltration of adipose tissue and promotes inflammatory cytokine release in high fat diet fed rats. This phenotype is mediated by decreased expression of AMPK and SIRT1, key nutrient sensors that regulate adipogenesis, lipid storage, and inflammation [86]. Thus, the overall effect of low vitamin D/VDR activation is increased susceptibility to obesity and associated diseases.

In macrophages, VDR is post-translationally regulated by blood glucose levels. VDR is OGlcNAcylated in response to hyperglycemia [87]. Whether this post-translational modification occurs in other obesity related organs such as adipose tissue, pancreas, or skeletal muscle and what downstream effect it may have on insulin sensitivity has yet to be determined. However, phosphorylation and SUMOylation of VDR influences its transcriptional activity [88]. Thus, further study regarding how VDR responds to blood glucose levels in the immune system and metabolic tissues is warranted [88]. These studies should be done in a tissue specific experimental model to avoid the confounding changes in the whole body knockout mice.

## 6. Potential Gut-Liver Axis through VDR and Microbiota in Obesity

**Figure 1:**
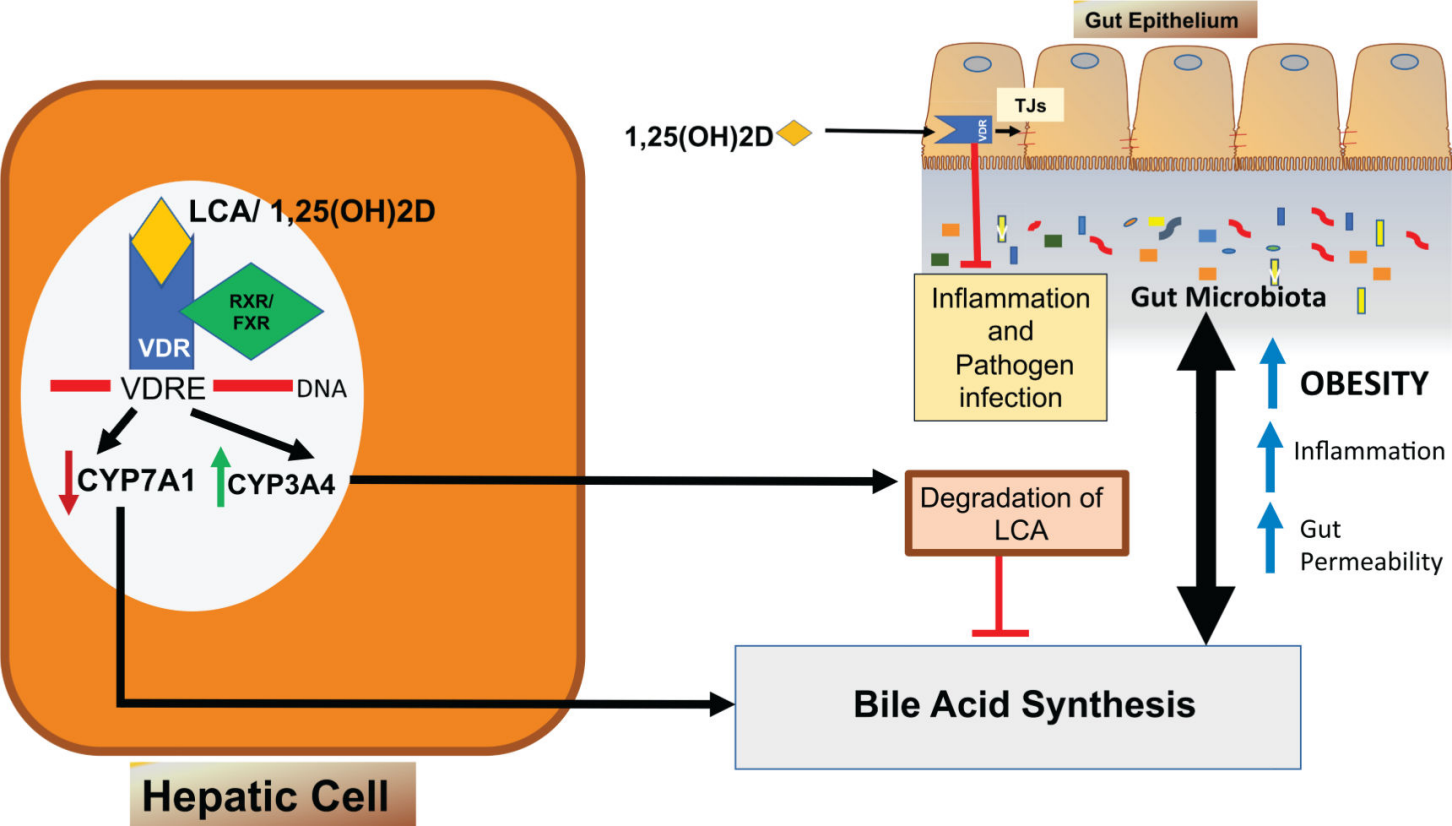
VDR regulates the liver-gut axis: In the liver, after VDR is activated by l,25(OH)2D or LCA, it then heterodimerizes with FXR/RXR and binds to VDR responsive element (VDRE) region of DNA. Active VDR directly increases CYP3A4 transcription, which in turn degrades LCA. VDR activation also induces negative regulation of CYP7A1, which decreases bile acid synthesis. Thus, VDR acts as a bile acid sensor. In the intestine, VDR regulates the intestinal barrier functions by regulating epithelial tight junction proteins and blocking inflammation and bacterial infection. Thus, VDR plays a major role in linking the signaling pathways that moderate the liver-gut axis. Dysregulation of this axis might lead to increased gut permeability, bacterial translocation, and obesity.

VDR, as a transcription factor, heterodimerizes with the retinoid X receptor (RXR) and regulates a range of physiological functions [89]. It may mediate the effects of the host microbiota by serving as a bile acid sensor [53]. VDR is activated by binding to either the potent vitamin D metabolite 1,25(OH)_2_D_3_ or secondary bile acids transformed by gut microbial metabolism, such as lithocholic acid (LCA), glycine-conjugated LCA, and 3-keto-LCA from 7α-dehydroxylated primary CDCAA [90]. When activated, VDR directly enhances the transcription of cytochrome P450 enzyme CYP3A4 in hepatic cells. CYP3A4 initiates the metabolic degradation of LCA and ultimately reduces the bile acid concentration, thus preventing bile acid toxicity (Figure 1). In the liver, after VDR is activated by l,25(OH)2D or LCA, it then heterodimerizes with FXR/RXR and binds to VDR responsive element (VDRE) region of DNA. VDR activation decreases bile acid synthesis. Thus, VDR acts as a bile acid sensor. Recently, Ishizawa *et al.* found that, like l,25(OH)2D3, LCA can also effectively increased the expression of target gene Cyp24a1 in the ileum, thus indicating that LCA is a selective VDR ligand acting mainly in the lower intestine. Correspondingly, LCA may be involved in signaling that interlinks intestinal bacteria and host VDR function [91]. VDR activation also creates a negative feedback loop by decreasing bile acid synthesis, mediated by reduced CYP7A1 expression (Figure 1). In male Sprague-Dawley rats, VDR has been shown to induce apical sodium-dependent bile acid transporter (Asbt) expression, which increases ileal bile acid transport [92].

As shown in Figure 1, in the intestine, VDR regulates the intestinal barrier functions by regulating epithelial tight junction proteins and blocking inflammation and bacterial infection. Thus, VDR plays a major role in linking the signaling pathways that moderate the liver-gut axis. Activation of VDR has multiple effects on bile acid metabolism, transport and absorption. VDR activation also directly interferes with the production of pro-inflammatory cytokines [93]. Dysregulation of vitamin D/VDR might lead to increased gut permeability, bacterial translocation, and obesity.

The Vitamin D/VDR axis plays a crucial role in obesity, which is at least partially mediated by gene polymorphisms and inflammation [94]. Results of a genome-wide association analysis of a Northern Germany cohort involving 1,812 human subjects suggest that the *Vdr* gene is significantly associated with overall microbial variation and individual taxa [78]. VDR regulates not only gut microbes but also several important host pathways, such as nucleotide-binding oligomerization domain-like receptors (NLRs), amino acid and carbohydrate metabolism, detoxification, infection, and signal transduction and influences cancer and other diseases [62]. A recent study of 103 pediatric patients in the UK showed that polymorphisms in the vitamin D metabolic pathway are associated with increased steatosis and severity of non-alcoholic fatty liver disease (NAFLD). Additionally, this NAFLD patient cohort exhibited decreased vitamin D status year-round with extremely low levels in the winter season [95].

A growing body of evidence suggests that microbiota-bile acid interactions play a role in the development of obesity and several other metabolic disorders [11, 96, 97]. Thus, the crosstalk between intestinal VDR signaling and gut microbiota may hold the key for how VDR signaling in non-adipose tissues can contribute to obesity and metabolic syndrome. Thus, a gut-liver axis mediated by VDR may play a novel role in obesity and metabolic diseases (Figure 1).

## 7. Potential Clinical Application of Probiotics and FMT in Prevention and Treatment of Metabolic Syndrome

Lately, targeting the microbiome to combat obesity and metabolic syndrome has gathered much attention. To modify the microbiome and modulate pathogenic bacterial species, prebiotics [98], probiotics [99], phage therapy [100] and CRISPR/cas [101] systems are evolving as advanced tools.

Several studies have indicated that administration of probiotics has clinical benefits in improving the symptoms of metabolic syndrome. Probiotics can promote restoration of gut microbiome composition by introducing live bacteria or bioactive metabolites [102, 103]. They also known to alter various metabolic pathway outcomes, most importantly, amino-acid metabolism, methylamines and SCFAs, suggesting differences in fermentation patterns [83]. Table 1 and Table 2 summarize the effect of probiotic bacteria in obesity in both mice and humans. *Lactobacillus gasseri, L. salivarius* UCC118, and a mix of the probiotic strains of Bifidobacterium were able to reduce adiposity and body weight in experimental animals/human groups as well as limit disease progression by countering the adverse effects of the high-fat diet that is often seen in patients suffering from the disease [6]. The VSL #3 probiotic combination has been shown to improve glucose tolerance by stimulating the production of GLP-1 and SCFAs, especially butyrate, which in turn help in reducing food intake and adiposity [53]. Furthermore, many probiotic strains such as *Lactobacillus rhamnosus* GG, L. *rhamnosus* Lc705, and *Saccharomyces boulardii* have been successful in clinical trials and are used for altering the composition of the host microbiome [104–106].

Alteration of microbiota to confer health benefits can be achieved by fecal microbiota transplantation (FMT). FMT is defined as the administration of a solution of fecal matter from a healthy donor into the intestinal tract of a recipient to directly change the recipient’s microbial configuration. FMT has proven to be effective in treating *Clostridium difficile* infection. There are also encouraging data in intestinal disorders like IBD, IBS, and MetS [107]. Fecal transplant from captive non-human primates on a low-fiber diet to GF mice induced obesity in the recipient mice, indicating that the composition of gut microbiota, along with fiber and diet composition, can potentiate the disease state [108]. In another report, IR in high-fructose diet fed adult rats was reversed by FMT from their control group [109]. Additionally, after high fat or high fat/high sucrose diet-fed obese mice underwent FMT from resveratrol-fed donor mice, they exhibited improved glucose homeostasis with increased anti-inflammatory markers, indicating diminished symptoms of obesity [110]. Although FMT results are encouraging in animal models, there are only 11 clinical trials supporting FMT as a potential therapy for MetS and obesity. Also, Vrieze *et al.* [77] reported that when obese patients diagnosed with T2DM received FMT from healthy/lean donors there were no significant changes in BMI or insulin sensitivity, and no alteration in microbial diversity was noted.

Prebiotics, probiotics, and FMT techniques are becoming more attractive potential therapies to treat microbial dysbiosis and metabolic diseases due to their few side effects - no major consequences have been reported so far. However, more detailed research needs to be done with proper standards and assessment to further establish the direct role of the microbiota in diseases. Different bile acid receptors - at least four members of the NR superfamily (FXR, PXR, CAR and VDR) - are therapeutic targets for the treatment of cholestatic liver disease, fatty liver disease, diabetes mellitus, and metabolic syndrome [53,111]. The application of targeting nuclear receptors, such as VDR, and the microbiome in therapy warrants further exploration.

## 8. Conclusion

**Figure 2:**
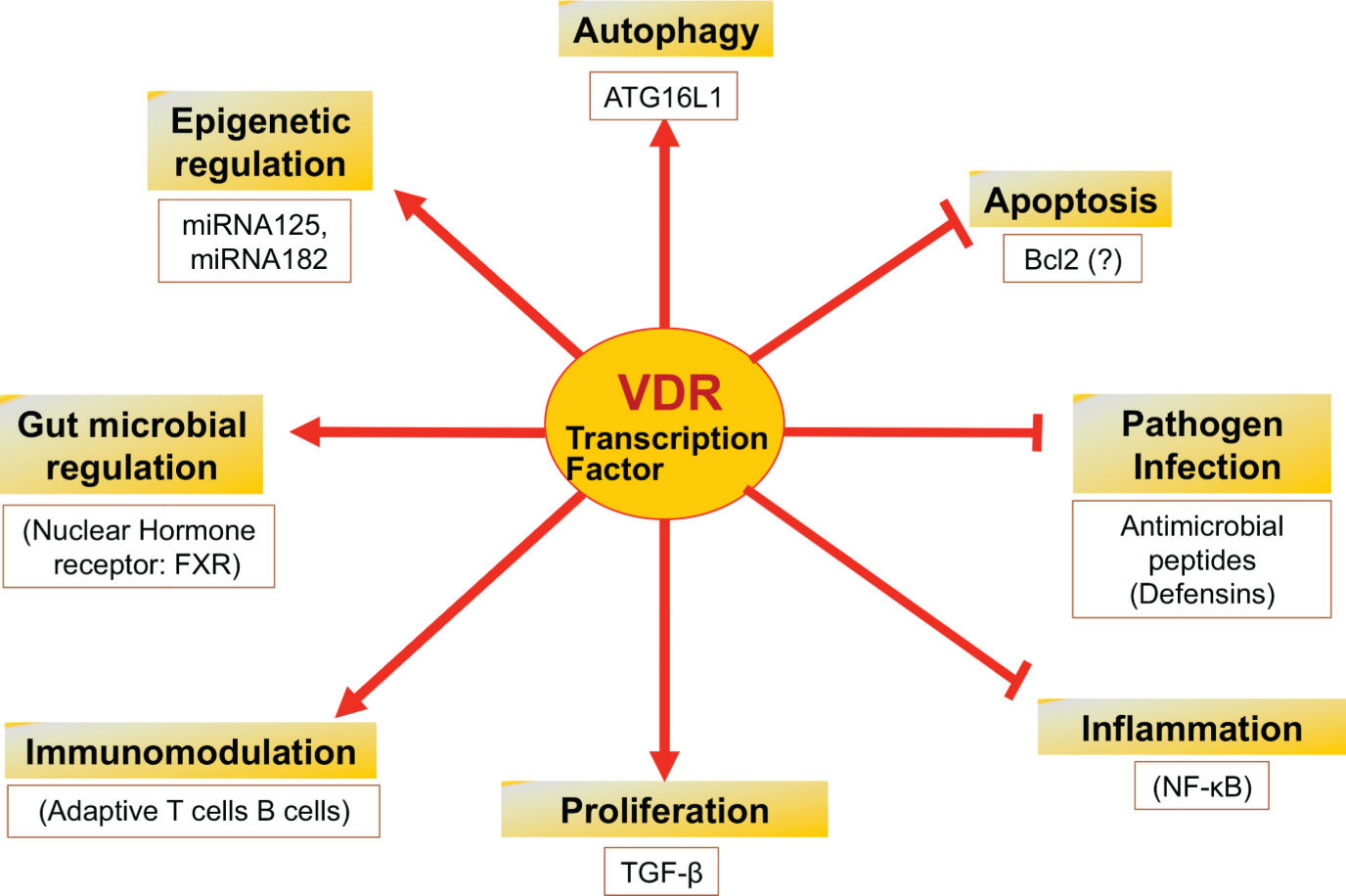
Vitamin D receptor in regulating various cellular and molecular functions: VDR regulates many vital physiological functions including immuno-modulation, proliferation, and autophagy via distinct effector molecules (as mentioned in the respective box). It directly influences the gut-microbiome and is involved in epigenetic modulation of the host. VDR is also known to block pathogen infection by regulating Paneth cells and anti-microbial peptides, such as defensins.

Dietary vitamin D is critical for a healthy state. VDR is involved in many important functions, including immunomodulation, proliferation, and autophagy via distinct effector molecules (Figure 2). The status and function of VDR directly influences microbiome and is involved in innate immunity and epigenetic modulation of the host. Vitamin D/VDR deficiency is a health concern in metabolic diseases. Recent progress of microbiome and vitamin D/VDR in inflammation and disease are very encouraging. A link between VDR and microbial metabolites has been established, but studies linking nuclear receptors, microbiome, and FMT are still lacking.

The research progress of microbiota regulated by Vitamin D receptor is an example of the critical role of nuclear receptors. Similar to the VDR, other nuclear receptors, such as the aryl hydrocarbon receptor, also regulate intestinal microbiome, enhance barrier integrity, reduce inflammation and autoimmunity, and increases disease tolerance. Tryptophan metabolites may suppress inflammatory responses by acting on the aryl hydrocarbon receptor in T-cells or astrocytes [112–115]. Further elucidating the mechanisms for microbiota and nuclear receptors will provide new opportunities to develop novel approaches that prevent and treat metabolic diseases.

## Figures and Tables

**Table 1:** Effects of probiotic bacteria in obesity in animal models.

Probiotic Bacteria Used	Study Model (Animal)	Major Findings and Effect on Gut microbiota (if any reported)	Reference
*Lactobacillus mali* APS 1	HFD-fed mice	↓ BW (body weight), caloric intake and fat accumulation. Gut microbiota restored.	Chen et al., 2018 [116]
B. *lactis* Bil, B. *breve* Bbr8 and *B. breve* BL10 mixture	HFD-fed mice	Prevents the progression of obesity, reduces adiposity and inflammation and improves lipid profile.	Roselli et al., 2018 [117]
*L. plantarum* HAC01	C57BL/6J HFD-fed mice	Improved insulin sensitivity and gut permeability ↓ LPS production.	Park et al., 2017 [118]
*L. rhamnosus* NCDC17	HFD and streptozotocin treated Wistar rats	↑ Insulin Sensitivity, glycosylated hemoglobin, FFA, TGs Improved lipid profile and oxidative stress. ↑ GLP1 and adiponectin, ↓ Inflammation by decreasing pro-inflammatory cytokines and propionate (in caecum)	Singh et al., 2017 [119]
*Probiotic mixture (L. salivarius 33, L. rhamnosus* LMG S-28148, *B. animalis subsp. lactis* LMG P-28149*)*	C57BL/6J HFD-fed mice	↓ BW and adiposity with improvement in insulin resistance, ↓ adipose tissue inflammation, ↑ dyslipidemia through adipose tissue immune cell-remodeling	Alai’d et al., 2016 [120]
*Lactobacillus sakei* OK67	HFD-fed mice	↓ BW and epididymal fat, NF- κB activation, LPS production, and pro-inflammatory cytokines	Lim et al, 2016 [121]
*Lactobacillus paracasei* CNCM 1–4270 (LC), *L. rhamnosus* 1–3690 (LR) and *Bifidobacterium animalis* subsp (BA)	HFD-fed mice	Each strain changed a distinct set of microbial population. LC and LR increased cecal acetate levels. BA significantly decreased adipose and hepatic tumor necrosis factor-α gene expression. Attenuates obesity.	Wang et al, 2014 [122]
*L. coryniformis* CECT5711	C57BL/6J HFD-fed mice	Improve insulin sensitivity and gut permeability ↓ LPS production.	Toral et al., 2014 [123]
*L. casei* NCDC 19	C57BL/6J HFD-fed mice	↓ BW, epididymal fat, blood glucose, plasma lipids and leptin f adiponectin	Rather et al., 2014 [124]
VSL#3	C57J/B67 HFD-fed and *ob/ob* mice	↓ adiposity	Yadav et al., 2013 [125]
*L. salivarius* UCC118	C57BL/J6 HFD-fed mice	↓ BW	Clarke et al., 2013 [126]

**Table 2:** Effects of probiotic bacteria in obesity in human studies.

Probiotic Bacteria Used	Study Model (Human)	Major Findings	Reference
Dietary supplementation with probiotics	Dietary supplementation with probiotics/ placebo controlled	Significantly alters the levels of DNA methylation in 38 obesity related genes in mothers and 68 genes in children.	Vahamiko et al, 2018 [127]
*L. salivarius* UBL S22 and Prebiotic Fructoligosaccharide in healthy human volunteers	Double-blind, placebo controlled	Improvement in serum TGs and lipid profile ↓. inflammatory cytokines	Rajkumar et al., 2015 [128]
Probiotic (*S. thermophiles, L. plantarum, L. acidophilus. L. rhamnosus. B. lactis. B. longum and B. breve)* and *Bofutsushosan* herb	Double-blind, placebo controlled	↓ BW, waist circumference and Fipid profile (in HDF-cholesterol). Change in body composition is positively related to levels of EPS and *L. plantarum*	Lee et al., 2014 [129]
